# Designing the community training hub pilot as a model for multidisciplinary workforce development

**DOI:** 10.3389/fmed.2025.1518625

**Published:** 2025-03-10

**Authors:** Sarah-Anne Munoz, Adalia Ikiroma, Rachel Erskine, Trish Gray

**Affiliations:** National Centre for Remote and Rural Health and Care, NHS Education for Scotland, Edinburgh, United Kingdom

**Keywords:** training, workforce, rural, evaluation, collaboration

## Abstract

The Community Training Hub (Hub) Pilot aims to contribute towards addressing recruitment and retention challenges in Scotland’s primary care workforce, with a particular applicability to the Remote, Rural and Island (RRI) context. A mixed methods evaluation framework has been designed to assess the effectiveness of the Hub multidisciplinary training across healthcare teams. The pilot involves General Practitioners (GPs), Advanced Nurse Practitioners (ANPs), Pharmacists, and Practice Nurses. This paper outlines the evaluation methodology, focusing on skill development, retention, and collaborative care. The paper argues for further evaluation of the Hub model to assess its potential as a model of distributed training and education to enhance workforce sustainability in rural healthcare.

## 1 Introduction

Scotland’s National Health Service (NHS) operates within a publicly funded healthcare system, providing universal access to care, free at the point of delivery. This system is underpinned by the principle of equitable healthcare access, which poses unique challenges in geographically remote rural and island (RRI) areas due to low population density, geographic isolation, and higher healthcare delivery costs. Unlike healthcare systems reliant on private insurance, Scotland’s NHS must allocate resources to meet the needs of diverse population within a constrained budget, which can amplify the impact of workforce shortages and access barriers in RRI settings.

RRI regions in Scotland face several persistent healthcare challenges, including workforce shortages, an ageing population, and increasing patient complexity. Stakeholders participating in range of qualitative interviews around primary and secondary care in R&R Scotland identified “R&R recruitment and retention” as the top priority (1). The recruitment and retention of healthcare professionals in RRI primary care settings has been a significant challenge across many countries (2). Another key issue is ensuring that small and often disparate teams receive the amount and level of training and education that they need to feel prepared for rural practice which can include increased generalist skills, given the high numbers of patient contacts and particularly ageing populations with high co-morbidities (3, 4).

These challenges require innovative solutions to improve accessibility, quality, and sustainability. Recognising these issues, the Scottish Government has implemented several policy measures to try and strengthen the healthcare workforce. For example, the Transforming Roles programme, introduced in 2017, focused on advanced practice roles within nursing, midwifery, and health professions to optimise service delivery (5, 6). Similarly, the 2018 Scottish General Medical Services (GMS) contract emphasises the GP’s role as a “senior clinical decision maker” and “expert medical generalist” within extended multidisciplinary teams (MDTs) (6, 7) to enhance primary care delivery. However, RRI GPs encounter unique challenges, highlighting the need for tailored solutions to support these practitioners and address complex health inequities in rural settings.

Globally, rural, and remote healthcare faces common challenges, including recruitment and retention of skilled professionals, limited access to education and training, and disparities in healthcare outcomes. While the specifics vary by region, high-income countries like Scotland share common concerns with other nations, such as the need for distributed education models to address geographic barriers and sustain a resilient healthcare workforce. Initiatives, such as the Rural Training Track (RTT) in the United States and Continuing Medical Education (CME) initiatives in Canada and Australia have successfully improved rural workforce capacity through strong stakeholder collaboration (8, 9, 24). These efforts emphasise addressing challenges such as long distances and drive times to urban centres where in-person training is delivered and difficulties with finding appropriate staff cover to allow others to attend training. Harnessing digital health technologies means to deliver distributed education can help, therefore, meet the continuing professional development needs of RRI primary care practitioners (10, 11). The use of online training and other digital educational strategies have been shown to help increase access to training and education for R&R primary care practitioners. Moreover, increased support for RRI generalist training will translate into a greater percentage of the workforce possessing the skills required for R&R practice (12, 13).

A Community Training Hub (the Hub) Pilot has been designed in Scotland to improve workforce recruitment and retention through multidisciplinary training and education which would be accessible and relevant for RRI healthcare teams. The Hub pilot initiative was designed by a group of stakeholders through collaborative co-development with two practices serving R&R populations and one serving an urban population. The initial pilot phase aims to support General Practitioners (GPs), Advanced Nurse Practitioners (ANPs), Pharmacists, and Practice Nurses in their professional development. A Hub is defined as “any action taken to support the coordination of education, learning and development that focuses on planning and upskilling primary care and community health workforces” (14) with the goal of improving generalist and emergency care capacity in rural contexts (13).

Several studies emphasise the importance of stakeholder engagement in such training initiatives. Stakeholders, ranging from programme participants and trainers to policymakers and community leaders, have been seen to play a critical role in areas as diverse as routine data collection, evaluation, and shaping the effectiveness of programmes (15). Moreover, stakeholder engagement in the evaluation and collection of routine data for education and training initiatives is fundamental for ensuring the efficacy, relevance, and sustainability of these initiatives (16). Despite the existence of such strategies, there is limited comprehensive evidence on how to support the development and implementation of digital distributed education initiatives tailored specifically to the needs of RRI primary healthcare teams. Although the existing literature highlights the importance of interdisciplinary collaboration and the development of generalist skills to address workforce shortages in these settings, this has not yet been fully adopted in most RRI primary healthcare setting.

The paper argues for further evaluation of the Hub model to assess its potential as a model of distributed education to enhance workforce sustainability in rural healthcare and outlines a methodology for doing this. It also details the collaborative approach to the development of a Hub pilot in Scotland and outlines the protocol for its evaluation. By improving collaboration and skill development, the Hub aims to address critical workforce sustainability issues. NHS England have successfully implemented a Hub model to enhance interprofessional training for the whole primary care team backed by substantial funding (17). We hope that our future evaluation results will prove useful to the development of R&R primary healthcare policy in Scotland and elsewhere.

## 2 Methods

### 2.1 Workshop design and stakeholder engagement

The Community Training Hub (CTH) pilot was developed through a collaborative and participatory approach. Two virtual workshops (March and May 2024) engaged stakeholders, including GPs, Advanced Nurse Practitioners (ANPs), pharmacists, practice managers and practice nurses, to design the Hub. These workshops were held via Microsoft Teams, which served as the primary platform for engaging stakeholders in designing the Hub and building its evaluation framework. Each workshop lasted 2 h and included structured activities such as brief presentations, group discussions, and brainstorming sessions.

The workshops were attended by 23 participants: 11 in the first workshop and 12 in the second. Attendees comprised staff from NHS Education for Scotland (18, 19), the National Centre for Remote and Rural Health and Care (NC), and representatives from five GP practices located in urban, remote, and rural settings. Participants were eligible if they were healthcare trainers aged 18 or above, working in remote or rural GP training practices, and able to participate in the CTH activities. Stakeholders included GPs, Advanced Nurse Practitioners (ANPs), pharmacists, practice managers, and practice nurses.

Discussions during the workshops explored existing approaches to training, challenges in workforce development, educational and professional support needs, and potential contributions of GP practices to the CTH pilot. A key focus was on identifying gaps and barriers in training and exploring strategies for workforce sustainability, such as adopting a Train-The-Trainer (TTT) model and standardizing multidisciplinary training (20).

### 2.2 Data collection

Data were gathered through a combination of detailed notetaking, workshop recordings, and subsequent verbatim transcriptions. These methods ensured a comprehensive and accurate capture of discussions, allowing for an in-depth analysis of stakeholder perspectives. The workshop discussions were reflexive and interpretative, providing insights into both practical and theoretical considerations for the CTH pilot as a recruitment and retention strategy. Stakeholders engaged in the workshops contributed perspectives informed by their varying levels of involvement with the CTH. Some participants provided practical insights based on their direct experiences with training and education, while others considered the pilot’s potential application in broader contexts. These discussions shaped the evaluation framework, ensuring its relevance and applicability.

### 2.3 Data analysis

Thematic analysis of the workshop data was conducted using Braun and Clarke’s six-step framework (21–23). This approach acknowledges the researchers’ subjectivity and reflexivity while providing a structured interpretation of the data. The analysis involved:

1. *Data Familiarisation*: Reviewing recordings, transcripts, and notes to gain an in-depth understanding of the data.
2. *Code Generation*: Manually coding the dataset to identify meaningful segments of text.
3. Theme
4. *Identification*: Grouping codes into potential themes and iteratively refining these themes for coherence and accuracy.
5. *Reviewing* Themes: Ensuring that the identified themes accurately represented the dataset and provided meaningful insights.
6. *Defining Themes*: Clearly naming and defining the themes to reflect their significance within the context of the study.
7. Reporting: Integrating themes into a structured narrative supported by illustrative data extracts from the workshop discussions.

The thematic analysis was conducted by a single researcher, with periodic reviews to validate the interpretations and ensure consistency. No formal triangulation process was employed, but multiple reviews of the transcripts and researcher reflexivity contributed to the reliability of the analysis.

### 2.4 Evaluation framework

A realist evaluation model was chosen to assess the CTH pilot due to its ability to account for the complexity of healthcare systems. The realist approach focuses on understanding what works, for whom, in what contexts, and why. The decision to adopt this model was informed by the pilot’s objectives to evaluate not only participant satisfaction and knowledge acquisition but also the practical application of training in real-world scenarios and its impact on workforce sustainability. A logic model links training activities to short- and long-term impacts, including workforce retention and improved clinical capacity.

## 3 Results

The Hub Pilot workshops revealed key themes that shaped the design and implementation of the project. These themes informed both the training model and the evaluation framework.

### 3.1 Stakeholder feedback

The workshops underscored the importance of flexible staffing, particularly in R&R settings where diverse employment contracts and workloads can hinder training efforts. Participants emphasised the need for multidisciplinary collaboration and the creation of a standardised competency framework for ANPs to ensure consistency in care delivery across practices.

#### 3.1.1 Themes

##### 3.1.1.1 Identified

- *Support for Diversity*: Practices highlighted the importance of Multi-Disciplinary Teams (MDTs) and training approaches that accommodate the unique needs of their patient populations.
- *Flexible Staffing*: Participants noted that the scheduling of tutorials and clinical support for ANP trainees posed a challenge due to varied employment contracts. Addressing these barriers was seen as critical to improving retention and skill development.
- *Growth Opportunities*: The workshops identified the potential for the Hub to enable growth through team-based, interdisciplinary training that builds both clinical and collaborative skills.
- *Purpose-Driven Coalition*: A consistent theme was the need for standardisation in ANP training and the importance of national frameworks to guide competency development and improve workforce retention.

Participants expressed their uncertainties around what task was and was not appropriate practice in terms providing support for ANPs. There was a call for clear and nationally agreed standard.

*“For the individual, which is not necessarily ideal, you are trying to set up a community training hub with sort of standards, but a lot of it is about, you know, individual learning needs and for different practitioners and the speed that they come on. I do not think it’s a one-size fits all.” (GP).*

*“So, I suppose I wonder if there’s a standardised training material for nursing or you know whoever you are training that can be used you know consistently, I do not know how it works, but you know we do some inhouse tutorials using people’s knowledge like my expertise…” (GP Trainer).*

*“I was just going to suggest like a standardised competency framework that is across the whole like a national thing for specifically for kind of primary care, so that we are not, they are not being dictated to by boards etc., and are kind of formalised, whether that’s advanced nurse practitioners, advanced pharmacists, advanced paramedic practitioners, these kind of routes and it could incorporate maybe the first, I do not know year of the GP training.” (ANP).*

One participant expressed excitement at the term “standardised,” highlighting the agreement among all participants, and said: “*I think all of us from the four different areas are sort of… the word standardised seems to be sort of recurrently repeated, and I think that’s definitely something that we have experienced you know…, and so, you know I suppose that [standardisation] would help all of us.” (GP Trainer)*

### 3.2 Outcomes

#### 3.2.1 Collaborative MS teams space

The earlier workshops facilitated the creation of a centralised digital space, using Microsoft Teams. The practices agreed on the importance of the collation of resources to see what was being used and to allow an opportunity to start standardising training and educational content across the practices thereby supporting the training and education of the MDTs. In addition, this Microsoft Team’s channel could be used as community space and to give an opportunity to primary care practitioners for peer support/education/discussions, that they might not otherwise have had the opportunity to do so. The MS Teams space provides an environment for knowledge exchange and practitioners to ask questions. This has an aim of addressing issues in primary care that can be particularly acute in remote and rural locations, such as professional isolation and lack of peer support.

#### 3.2.2 The Hub in Turas

The Hub is in development and hosted on Turas; Turas is the national, digital learning management environment developed by NHS Education for Scotland to support health and care professionals working in the public sector. All staff within the three primary care practices involved in the pilot have access to Turas. The Hub will therefore be a repository for knowledge exchange, education, and training resources, to support practitioners to provide safe and effective primary care provision in RRI communities. Working collaboratively the design of the Hub in Turas was undertaken by staff working in the National Centre for Remote and Rural Health and Social Care incorporating the four pillars of practice identified by NES (undated) - clinical practice; facilitation of learning; leadership; and evidence – research and development. These basic principles and values are consistent among the professional groups involved in the pilot; in addition, specific training and education resources that are aimed across these professional groups have been collated as well as those with a specific remote and rural focus. Collation of the resources has been led by one of the Advanced Nurse Practitioners across the three practices who was supported by the practices involved. This collation of resources by the ANP was then returned to the National Centre staff using the MS Teams space as shown below to be uploaded to Turas. Feedback on the design, layout, and resources themselves was collated using MS Office forms during development.

#### 3.2.3 Stakeholder-informed evaluation framework

The workshops also facilitated the creation of an agreed evaluation framework. Implementation of this framework will help address the lack of evidence on digital Hubs as a distributed education initiative for RRI workforce.

It was agreed that the evaluation of the Hub Pilot would employ a concurrent explanatory mixed methods design, integrating quantitative and qualitative data to evaluate training effectiveness. The evaluation framework developed is grounded in the principles of realist evaluation, i.e., focusing on interactions between context, mechanisms, and outcomes.

##### 3.2.3.1 Quantitative data collection

Structured surveys and assessments will gather data on participants’ demographics, pre- and post- training knowledge, skills development, retention rates, and economic contributions.

##### 3.2.3.2 Quantitative key performance indicators (KPIs)

To assess the effectiveness of the Hub Pilot, a range of quantitative metrics were selected to measure participant engagement, knowledge acquisition, and workforce retention. These KPIs provide valuable insight into how well the multidisciplinary training has impacted professional development and long-term retention of healthcare professionals in RRI settings:

- Number of training sessions and attendance rates.
- Pre and post training knowledge assessments.
- Retention rates of trained healthcare professionals.

##### 3.2.3.3 Qualitative data collection

In-depth interviews and focus groups will explore participant experiences, stakeholder engagement, and adaptability of the Hub pilot.

###### 3.2.3.3.1 Qualitative themes

In addition to quantitative metrics, qualitative data will be collected through interviews and focus groups. Thematic analysis of these sessions will involve predefined and emergent coding; predefined themes relating to perceived effectiveness of the Hub, stakeholder engagement, and programme adaptability to local needs. Emergent codes will also offer a deeper understanding of participant experiences:

- Perceived effectiveness of the Hub.
- Stakeholder collaboration.
- Programme adaptability to community needs.
- Emergent themes.

##### 3.2.3.4 Integration of data

Quantitative and qualitative data will be integrated for cross-validation, ensuring a comprehensive evaluation of the pilot’s impact on professional development and workforce retention.

##### 3.2.3.5 Evaluation logic model

The following logic model outlines the key resources, activities, outputs, and impacts for the Hub Pilot. It serves as a roadmap, showing how the inputs of the pilot lead to both short- and long-term outcomes. The model was developed with stakeholder input to ensure the pilot meets the specific needs of R&R PC teams and ultimately improves workforce retention and clinical capacity.

**Table 1:** 

Resources/Inputs	Activities	Outputs	Outcomes	Impacts
NES/NC: Hub platform, expertise, funding, network. HUB Testers: Trainers, materials, resources, trainees.	Conduct training for MDTs. Workshops, collaborative core training plans. Develop learning opportunities.	Expanded training. Refined learning schedule. Increased identification of retention challenges.	*Short-Term*: MDTs engaged *Mid-Term*: MDT knowledge growth *Long-Term*: Nationwide training support through centralised platform; workforce development through long-term creation of career growth opportunities by fostering skill advancement and continuous learning initiatives.	Improved workforce retention and clinical capacity in R&R PC services.

The MDTs will participate in regular training within their practice and other training organised through the hub whiles using available resources independently for their professional development. Their engagement will be crucial for the success of the pilot, requiring active involvement from all participating MDTs. The MDTs are expected to grow both in terms of expertise and coordination of patient care. This will involve targeted skill development through ongoing training and cross-functional collaboration, allowing MDTs to take on new clinical pathways and expand their leadership roles. This, in turn, will result in increased retention and recruitment of the workforce.

##### 3.2.3.6 KPIs across professional groups

The evaluation of the Community Training Hub Pilot utilises a comprehensive set of Key Performance Indicators (KPIs) to assess the effectiveness of multidisciplinary team (MDT) training across various healthcare professionals. These KPIs are designed to measure the impact of the training on knowledge acquisition, skill development, engagement, and overall workforce performance. Additionally, the evaluation includes metrics related to collaboration and partnerships, reflecting the importance of cross-disciplinary cooperation in R&R primary care settings. The table below outlines the key KPIs across different professional groups involved in the Hub Pilot:

**Table 2:** 

Profession	Training delivery KPIs	Engagement and satisfaction KPIs	Impact on knowledge and skills KPIs.	Collaboration and partnerships KPIs
Doctors	Number of Clinical Training Sessions and CME* Credits specific to R&R PC*.	Attendance Rate and Satisfaction Scores.	Pre- and Post-Training Assessment Scores and Practical Skill Demonstration.	Partnerships with Educational Institutions and Joint Courses.
Nurses	Number of Nursing Training Sessions, CEUs* specific to R&R PC.	Engagement Levels and Feedback Scores.	Knowledge Gain Metrics and Clinical Competency Skills.	Collaborative Training Initiatives and Partnership Impact.
Pharmacists	Number of Pharmacist Training and Certification Opportunities specific to R&R PC.	Participation Metrics and Survey Results.	Pre- and Post-Training Assessment Scores and Credentialling or Certification Achievement Rates.	Partnership with Pharmacy Schools and Collaborative Sessions.
ANPs	Number of Advanced Practice Sessions and Certification Opportunities specific to R&R PC.	Attendance Metrics and Satisfaction Ratings.	Advanced Skills Assessment and Certification Achievement Rates.	Advanced Practice Collaborations and Joint Certification Courses.
MDT	Total Training Sessions Conducted in R&R PC. Diversity of Training Courses in R&R PC. Attendance Rates.	Participation Rate and Satisfaction Survey Results. Team Dynamics Qualitative Feedback.	Assessment Scores, Application of Skills and Collaborative Case Studies.	Number of collaborative sessions, partnerships with educational institutions and joint training courses.

*CME, Continuing Medical Education; CEUs, Continuing Education Units; PC, Primary Care.

The plan is that the KPI data will be collected at three time points: T0 (at the start of the CTH pilot), T1 (at 12 weeks), and T2 (at 6 months), and T3 (at 9 months) using a one-group pre-test and repeated post-test design analysis methodology to determine the effectiveness of the CTH and track participants’ journey through the CTH pilot. Likert-Type Scales will be explored for all the outcome assessments. The KPIs were created based on our expertise in programme evaluation and scoping of existing literature on educational interventions and the metrics that were most relevant to the different professional groups. The selected KPIs provide clear insights into the critical success factors for each professional group’s performance. The core indicators for the evaluation would be Participant success rates, Retention rates post-training and Economic indicators (e.g., employment creation), and yes, the idea is to go back to the logic model to measure progress. That is, the number and percent of participants who use the CTH platform to support their learning needs, who report that they have increased knowledge, learnt new skills to support patient care, who do not have the intention to leave their role following completion of the pilot are indicators of how well the CTH is doing with respect to the outcome.

The evaluation of the Hub Pilot is designed to capture a wide range of outcomes, from short-term improvements in communication and skills application to long-term impacts on workforce retention and patient care quality. The table below outlines the expected short, intermediate, and long-term outcomes for each professional group involved in the pilot, highlighting how the training contributes to workforce stability, improved team dynamics, and enhanced patient outcomes in remote and rural (R&R) primary care settings.

**Table 3:** 

Category	Short-term outcomes	Intermediate outcomes	Long-term outcomes
Doctors	Workforce Development, Health Outcome Correlation	Team Performance Metrics, Patient Care Coordination	Recruitment and Retention Metrics, Workforce Development, Workforce Capacity
Nurses	Retention Rates, Patient Care Quality	Patient Satisfaction Scores	Retention and Patient Care Quality
Pharmacists	Professional Growth, Medication Error Rates	Team Performance Metrics, Patient Satisfaction Scores	Reduced Medication Errors, Professional Growth
Advanced Nurse Practitioners (ANPs)	Workforce Stability, Certification Advanced Care Delivery	Patient Care Coordination, Improved Care Quality	Retention and Job Satisfaction, Advanced Care Delivery
Multidisciplinary Team (MDT)	Improved Communication, Role Clarity, Immediate Skill Application, Turnover by Role	Team Performance Metrics, Improved Patient Care Coordination, Patient Satisfaction	Workforce Stability, Sustainable MDT Practices, Improved Patient Health Outcomes

## 4 Discussion

The Hub pilot represents an innovative approach to addressing workforce challenges in RRI healthcare by leveraging distributed education and multidisciplinary collaboration. Through stakeholder engagement, we have designed an associated evaluation framework. Through implementation of both, we aim to test the potential of the Hub as a scalable model for addressing the recruitment and retention challenges in R&R healthcare. By integrating tailored training into daily clinical practice, the HUB aims to foster skill development and a stronger sense of community among healthcare professionals, contributing to improved workforce stability.

The Hub pilot’s design and evaluation directly address its primary aim: to create a scalable and sustainable model for multidisciplinary training that supports workforce development in RRI settings. By engaging stakeholders, including GPs, Advanced Nurse Practitioners (ANPs), pharmacists, and practice managers, the pilot sought to develop an inclusive framework that reflected the realities of rural healthcare delivery. The emphasis on stakeholder-driven co-design ensured that the Hub model aligned with the practical and theoretical needs of the healthcare teams involved.

Stakeholder engagement during the design phase revealed key themes that directly informed the Hub’s structure. Stakeholders emphasised the need for flexible staffing, standardised training materials, and enhanced support for multidisciplinary teams, particularly ANPs. This is consistent with previous studies highlighting the importance of tailored education models in rural contexts (10, 13). These findings align with global strategies that emphasise distributed education and stakeholder collaboration to address geographic barriers to training (8, 9). The engagement also reinforced the need for shared ownership of the training model, ensuring its relevance and sustainability in these settings.

The evaluation framework developed for the Hub pilot is grounded in realist principles, which will add depth to the assessment of training outcomes. Unlike traditional models that focus solely on participant satisfaction, the realist approach examines how specific contextual factors influence the success of the intervention. This focus on “what works, for whom, and in what contexts” provides actionable insights for scaling the Hub model to other settings.

The KPIs outlined in the evaluation framework provide a detailed and structured approach to monitoring the effectiveness of the Hub Pilot. These metrics will track key areas such as knowledge acquisition, skill development, and multidisciplinary collaboration. For example, the Training Delivery KPIs highlight the diverse range of sessions tailored to specific healthcare professionals, ensuring that the learning opportunities are both relevant and applicable across different roles within remote and rural (R&R) primary care. By including participation and engagement metrics, the KPIs also ensure that the training’s reach and accessibility will be monitored, allowing for adjustments where necessary to maximise impact.

Collaboration and partnerships are central to achieving Hub success. By fostering connections between healthcare teams, educational institutions and other stakeholders, the Hub model may address resource constraints as often encountered in RRI settings. The hub’s emphasis on collaborative care is particularly important in rural contexts, where multidisciplinary teamwork is essential for positive patient outcomes.

The projected outcomes of the Hub pilot demonstrate its potential impact on workforce retention and patient care quality. Short-term outcomes, such as improved communication and role clarity within multidisciplinary teams, are crucial for ensuring immediate practical application of skills gained through training. Over time, these improvements are expected to lead to more substantial impacts, including better patient care coordination, reduced workforce turnover, and increased capacity within RRI healthcare. By addressing both immediate and systemic challenges, the Hub model aligns with broader goals of healthcare equity and sustainability.

### 4.1 Implications for policy

The Hub Pilot also has significant implications for policy. If successful, it could serve as a scalable model for similar initiatives across Scotland and beyond. By leveraging digital platforms like Turas and in-person workshops, distributed education through a Hub model suggests distributed education could bridge geographic barriers and support ongoing professional development in remote settings. This would align with international models of rural healthcare training, such as Australia’s Rural Training Track and Canada’s CME programmes, which have shown long-term benefits for workforce sustainability.

Future research will be essential to fully understand the Hub pilot’s impact. Longitudinal studies could explore how training interventions influence patient outcomes, workforce retention and healthcare system capacity over time. Additionally, further engagement with policymakers and healthcare providers will be crucial for scaling the HUB model and integrating it into national strategies for RRI healthcare improvement. While the study provides valuable insights, it is limited by its reliance on self-reported data from workshop participants, which may introduce bias. The absence of formal triangulation in the thematic analysis is another limitation that should be addressed in future studies.

## 5 Conclusion

From the viewpoint of the researchers’, the Hub brings significant advantages in addressing a spectrum of challenges in RRI healthcare settings. It has the potential to help RRI areas with their ongoing challenges in attracting and retaining talent, as well as reduce social disparities and promote the growth of a sustainable health workforce which, in turn, could lead to equitable and quality health care delivery across RRI areas.

## Data Availability

The original contributions presented in the study are included in the article/supplementary material, further inquiries can be directed to the corresponding author.
